# Targeting Radiation Resistance in Oesophageal Adenocarcinoma with Pyrazinib-Functionalised Gold Nanoparticles

**DOI:** 10.3390/cancers16234007

**Published:** 2024-11-29

**Authors:** Simone Marcone, Jolanda Spadavecchia, Memona Khan, Gabriele Vella, Fiona O’Connell, Marzia Pendino, Meghana Menon, Claire Donohoe, Ravi Narayanasamy, John V. Reynolds, Stephen G. Maher, Niamh Lynam-Lennon, Breandán Kennedy, Adriele Prina-Mello, Jacintha O’Sullivan

**Affiliations:** 1 Department of Surgery, Trinity Translational Medicine Institute, Trinity St. James’s Cancer Institute, Trinity College Dublin, D08 W9RT Dublin, Ireland; fiona.oconnell@pennmedicine.upenn.edu (F.O.); menonm@tcd.ie (M.M.); claire.donohoe@tcd.ie (C.D.); ravin@tcd.ie (R.N.); reynoljv@tcd.ie (J.V.R.); maherst@tcd.ie (S.G.M.); lynamlen@tcd.ie (N.L.-L.); osullij4@tcd.ie (J.O.); 2 CNRS, UMR 7244, CSPBAT, Laboratoire de Chimie, Structures et Propriétés de Biomateriaux et d’Agents Therapeutiques Université Paris 13, Sorbonne Paris Cité, 93000 Bobigny, France; jolanda.spadavecchia@gmail.com (J.S.); memonakhan94@gmail.com (M.K.); 3 Laboratory for Biological Characterisation of Advance Materials (LBCAM), Trinity Translational Medicine Institute, Trinity College Dublin, D08 W9RT Dublin, Ireland; gvella@tcd.ie (G.V.); prinamea@tcd.ie (A.P.-M.); 4 UCD School of Biomolecular and Biomedical Science & UCD Conway Institute of Biomolecular and Biomedical Research, University College Dublin, D04 V1W8 Dublin, Ireland; marzia.pendino@ucdconnect.ie (M.P.); brendan.kennedy@ucd.ie (B.K.); 5 Nanomedicine Group, Department of Clinical Medicine, Trinity Translational Medicine Institute, Trinity St. James’s Cancer Institute, Trinity College Dublin, D08 W9RT Dublin, Ireland

**Keywords:** radioresistance, gold nanoparticles, metabolism, angiogenesis, targeted delivery

## Abstract

**Background/Objectives:** Only 20–30% of oesophageal adenocarcinoma (OAC) patients achieve a complete response to neoadjuvant chemo-radiotherapy for locally advanced tumours. Enhancing the response to radiation therapy is critical for improving outcomes in this aggressive cancer. Pyrazinib (P3) is a promising compound with radiosensitizing, anti-angiogenic, anti-inflammatory, and anti-metabolic properties. However, its limited solubility and bioavailability have hindered its therapeutic potential. Methods: To overcome these limitations, pyrazinib was conjugated with gold nanoparticles (AuNP-P3), creating a novel formulation designed to enhance solubility, maintain bioactivity, and enable targeted delivery to tumour sites. Results: In an isogenic model of OAC radioresistance, AuNP-P3 significantly reduced the surviving fraction following irradiation, demonstrating its radiosensitizing properties. It also reduced mitochondrial metabolism and modulated the secretion of inflammatory mediators in both in vitro models of OAC radioresistance and human ex vivo OAC tumour explants. Furthermore, AuNP-P3 exhibited potent anti-angiogenic activity, significantly inhibiting blood vessel formation in vivo using zebrafish embryo models. Conclusions: These results collectively confirm that P3, in its conjugated formulation with gold nanoparticles, retains its therapeutic properties, highlighting the potential of AuNP-P3 as a novel therapeutic radiosensitizer for oesophageal adenocarcinoma and supporting its further development for clinical applications.

## Simple Summary

Oesophageal adenocarcinoma (OAC), an aggressive cancer of the food pipe, often responds poorly to current chemo-radiotherapy, with only 20–30% of patients achieving a complete response. Improving tumour sensitivity to radiation could greatly benefit patient outcomes. This study explores a new approach using pyrazinib (P3), a compound previously shown to enhance radiation sensitivity in OAC. By combining P3 with gold nanoparticles (AuNP-P3), we created a formulation that improves delivery and retains P3’s cancer-fighting properties. Tests demonstrated that AuNP-P3 enhances radiation sensitivity in cancer cells, reduces tumour-supporting factors, and prevents blood vessel formation, which is needed for tumour growth. These results suggest AuNP-P3 as a promising, targeted treatment to boost radiation effectiveness in OAC, potentially leading to more successful treatment outcomes for patients.

## 1. Introduction

Oesophageal cancer is an aggressive disease with poor prognosis, ranking among the most malignant cancers globally, with over 0.6 million new cases and 0.54 million deaths in 2020, with a cure rate of less than 20% [1]. Neoadjuvant treatment for locally advanced tumours often involves chemotherapy alone or combination chemo-radiotherapy (neo-CRT) [2]. However, only about 30% of patients benefit from these treatments [3], with the remaining 70% experiencing toxicities without therapeutic gain, leading to delayed surgery and reduced overall survival. Radiotherapy, a major treatment modality for many cancers, including oesophageal cancer, aims to maximize tumour control while minimizing damage to surrounding tissues [4]. Yet, insufficient tumour control and relapse remain significant challenges [5]. Several biological mechanisms contribute to radioresistance, including dysregulated cellular energetics, angiogenesis, and inflammation [6,7,8,9,10,11]. Targeting tumour metabolism emerges as a novel approach to enhance radioresponse in oesophageal adenocarcinoma (OAC). Our previous work demonstrated that radioresistant OAC cells exhibit altered metabolic phenotypes compared to radiosensitive cells, with a higher rate of oxidative phosphorylation [8]. Moreover, ex vivo analysis of metabolic parameters in treatment-naïve tumour samples revealed elevated ATP5B, a marker of oxidative phosphorylation, in patients with poor neo-CRT responses [8]. Inflammation, closely linked to cellular metabolism, plays a pivotal role in OAC’s pathogenesis and treatment response [12,13]. Buckley et al. showed a positive correlation between the inflammatory secretome profile in OAC biopsies and real-time metabolic rates [14,15]. Additionally, components of the complement system, elevated in OAC patients with poor neo-CRT responses, underscore the role of inflammation in radioresistance [12]. Angiogenesis, critical for tumour response to radiation therapy, is intricately linked to cellular metabolism [10,16]. Anti-angiogenic agents may enhance radiosensitivity by normalizing tumour vasculature, thereby promoting radiation therapy response [16,17]. However, tumour vascular responses to radiation depend on various factors, including radiation dose and fraction, tumour size, and location [18].

To address radioresistance, we identified and patented pyrazinib (P3) as a novel radiosensitizer in OAC with anti-angiogenic, anti-metabolic, and anti-inflammatory properties [19]. However, its limited solubility and bioavailability pose challenges for clinical use. Combining pyrazinib with gold nanoparticles (AuNPs) presents a promising strategy for improving the solubility of P3 and targeted drug delivery [20,21]. In this study, we evaluated the impact of pyrazinib-functionalized gold nanoparticles (AuNP-P3) on OAC metabolism, inflammatory secretome, angiogenesis, and radiosensitivity using in vitro, ex vivo, and in vivo models. Our goal is to develop a formulation that preserves P3’s bioactivities and serves as a platform for future targeted delivery in OAC treatment.

## 2. Methods

### 2.1. Ethical Approval

To conduct the human ex vivo explant work, full ethical approval for this study was granted by the joint St James’s Hospital/AMNCH ethical review (SJH/TUH Joint Research Ethics: 31853185). For the in vivo zebrafish study, the intersegmental vessel assay screen was approved by the UCD Animal Research Ethics Committee under protocol number AREC-22-07-Kennedy prior to the commencement of the study.

### 2.2. Pyrazinib, Gold Nanoparticles, and Conjugated Compounds

Pyrazinib (P3) was synthesized by Celtic Catalysts (Dublin, Ireland) and Onyx Scientific (Sunderland, UK), and the compound was dissolved in 100% DMSO. Colloids of COOH-terminated PEG-coated AuNPs (from now on PEG-AuNPs or AuNPs only for convenience) were prepared under a similar synthesis previously described [22,23]. Briefly, 20 mL of chloroauric acid (HAuCl_4_) aqueous solution (2 mM) was added to 0.25 mL of dicarboxylic PEG and mixed by magnetic stirring for 10 min at room temperature. To this solution, 3 mL of aqueous 8 mM NaBH_4_ was added at once. The as-prepared PEG-AuNP solution was purified by centrifugation and dialysis to remove excess of not-conjugated dicarboxylic PEG. Colloid concentration was assessed by standard methods described previously [22,23].

Conjugation of P3 onto PEG-AuNPs (AuNP-P3): Pyrazinib (P3) was grafted onto PEG-AuNPs by an electrostatic method. Briefly, 3 mL of PEG-AuNPs (2 mM) was mixed with 200 µL of P3 (1 mM) and 0.5 mM Sodium Chloride (NaCl) at a pH of 9. The solution was stirred at room temperature for 24 h. After that, dialysis with dialysis membrane tubing Spectra/Por 3 (molecular weight cut-off 3500 Da, Serva Electrophoresis, Heidelberg, Germany) was carried out with continuous stirring (150 rpm) to achieve the final colloidal solution with a final P3 concentration of 10µM. The solution was then buffered at a pH of 7.4. All characterization measurements were carried out by adopting an orthogonal approach on three replicates of the same samples to ensure the reliability of the synthesis and reproducibility of the measurements, in line with previous studies [22,23]. Absorbance spectra were recorded and analyzed for all samples. These spectra were obtained by using a double-beam Varian Cary 500 UV-Vis spectrophotometer (Agilent, Les Ulis, France) in the spectral range of 200–900 nm. The concentration of AuNP-P3 (10 µM) and the timepoints for the experiments employed in this study align with those utilized in our previously published research involving P3 [14,19].

### 2.3. Electron Microscopy

Transmission Electron Microscopy (TEM) images were recorded with a JEOL JEM 1011 microscope operating (JEOL, Peabody, MA, USA) at an accelerating voltage of 100 kV. TEM specimens were prepared after separating the surfactant from the metal particles by centrifugation, as previously described [22]. Briefly, 1 mL of PEG-AuNPs was centrifuged for 20 min at a speed of 14,000 rpm. The upper part of the colourless solution was removed, and the solid portion was redispersed in 1 mL of water. Furthermore, 2 μL of this redispersed particle suspension was placed on a carbon coated copper grid, manufactured by Smethurst High-Light Ltd. (Bolton, UK) and marketed exclusively by Agar Scientific, and dried at room temperature. An average of 100 nanoparticles was accounted for nominal size measurement using JEOL software (https://www.jeol.com/products/scientific/nmr_software/ accessed on 3 August 2015).

### 2.4. Dynamic Light Scattering (DLS) and Zeta Potential Measurements

Hydrodynamic particle size distribution and zeta potential measurements of all samples were recorded using a Zetasizer Nano ZS (Malvern Panalytical, Malvern, UK). DLS measurements were conducted at a temperature of 25 °C at a backscattering angle of 173 degrees. The hydrodynamic diameter (Z-average) and the dispersity from cumulative analysis were obtained according to ISO22412 [24]. The results represent averages from at least 3 consecutive measurements, together with the corresponding standard deviation from each readout.

### 2.5. Nanoparticle Tracking Analysis (NTA) Measurements

NTA analyses of the two AuNPs samples were conducted using a NanoSight NS500 system (Malvern Panalytical, Malvern, UK) in light scattering mode using the EUNCL PCC-023 validation protocol [25]. The system is equipped with a 405 nm laser and operated using NanoSight 3.2 software. PEG-AuNPs and AuNP-P3 samples were incrementally diluted, in line with the Malvern Panalytical optimization protocol and as presented in the EUNCL PCC-023 SOPs, to achieve an optimal concentration of 20–100 particles per field acquisition (camera field) at a final working volume of 1 mL. A total of six videos of 60 s were recorded for each sample, the detection threshold (25–800 nm size region) during analysis was selected to ensure that only distinct nano-objects were analyzed, and the value was kept constant during the recording. In 60 s, approximately 1200 to 6000 particles are recorded for each acquired frame. Accuracy and reproducibility are achieved by the detection of at least 10 regions of interest per second. Therefore, 12,000 to 60,000 particles were tracked per technical repeat. Additionally, consecutive videos bring the tracking of particles between 72,000 and 360,000 particles within each sample measurement campaign. This is enabling the statistical reproducibility of the particle size and concentration readouts.

### 2.6. OE33P and OE33R Cell Lines

The OE33 oesophageal adenocarcinoma cell line was obtained from the European Collection of Authenticated Cell Cultures. The isogenic model of radioresistant OAC, OE33P (radiosensitive), and OE33R (radioresistant) cells was generated as previously described [9]. Briefly, OE33 cells were exposed to clinically relevant fractionated doses (2 Gy) of radiation, receiving a cumulative dose of 50 Gy, using an Xstrahl CIX2 irradiator (XSTRAHL Ltd., Walsall, UK). A clonogenic assay confirmed the enhanced survival of the radioresistant OE33 subline (OE33 R). In this study, the irradiation of OAC cells and human tissue explants was performed using a Xstrahl CIX2 irradiator (XSTRAHL Ltd., Walsall, UK), with an aluminum-filtered dose rate of 1.87 Gy/min at the 50 FSD.

### 2.7. Clonogenic Assay

OE33P and OE33R cells were seeded at the optimized densities of 3 × 10^3^ in 1.5 mL complete RPMI in triplicate in 6-well plates and were allowed to adhere overnight. Then, 24 h later, the cells were treated with 10 μM of P3, AuNPs, and AuNP-P3 or with a control (0.1% DMSO + 0.1% water). Another 24 h later, OE33P and OE33R cells were either irradiated at 2 Gy or mock-irradiated. Colonies were allowed to grow for 10 days, at which point they were fixed and stained with 0.05% crystal violet (25% methanol in dH_2_O) and allowed to air dry. Colonies consisting of 50 cells or more were counted using a colony counter (GelCount^TM^, Oxford Optronix, Oxford, UK). Plating efficiency (PE), the fraction of colonies formed by untreated cells, was calculated using the formula PE = No. colonies/No. cells seeded. The surviving fraction (SF), the number of colonies formed, expressed in terms of PE, was calculated using the formula SF = No. colonies/(No. cells seeded × PE).

### 2.8. Seahorse Analysis of Metabolic Profiles in OE33P and OE33R Cells

OE33P and OE33R cells were seeded in triplicate at a density of 11,000 and 13,000 cells/well, respectively, in 24-well cell culture XFe24 microplates (Agilent Technologies, Santa Clara, CA, USA). Then, 5 h later, once the cells had adhered to the bottom of the microplate, 10 μM P3, AuNPs, AuNP-P3, or the control (0.1% DMSO + 0.1% water) were added to the cells. Following 24 h treatment, the cells were either irradiated at 2 Gy or mock-irradiated. Following this, 24 h after irradiation, the media were collected and stored at −80 °C for multiplex ELISA. Fresh Dulbecco’s Modified Eagle’s medium (DMEM) supplemented with 10 mM glucose, 1 mM pyruvate, and 2 mM glutamine (Agilent) (pH 7.4) was added to the cells and incubated for 1 h at 37 °C in a CO_2_-free incubator. Seahorse Xfe24 analyser was used to assess multiple parameters, including OCR and ECAR metabolic profiles, basal respiration, ATP-linked respiration, maximal and reserve capacities, and non-mitochondrial respiration (Seahorse XF Cell Mito Stress Test Kit, Seahorse XFe24 Analyzer, Agilent Technologies, Santa Clara, CA, USA), as previously described [19]. All measurements were normalized to the cell number using the crystal violet assay.

### 2.9. Multiplex ELISA of Cell Supernatant

Supernatants from OE33R and OE33P cells generated for Seahorse analysis were used for Multiplex ELISA. The supernatant was processed according to the MSD multiplex protocol (Meso Scale Discovery, Rockville, MD, USA). To assess angiogenic, vascular injury, pro-inflammatory, cytokine, and chemokine secretions from OE33P and OE33R cell supernatants previously treated with 10 μM P3, AuNPs, or the vehicle control (0.1% DMSO + 0.1% water) for 24 h, a 54-plex ELISA kit separated across 7 plates was used (V-PLEX Assay Platform, Meso Scale Discovery, Rockville, MD, USA). This kit was used to quantify the secretions of CRP, Eotaxin, Eotaxin-3, FGF (basic), GM-CSF, ICAM-1, IFN-γ, IL-10, IL-12/IL-23p40, IL-12p70, IL-13, IL-15, IL-16, IL-17A, IL-17A/F, IL-17B, IL-17C, IL-17D, IL-1RA, IL-1α, IL-1β, IL-2, IL-21, IL-22, IL-23, IL-27, IL-3, IL-31, IL-4, IL-5, IL-6, IL-7, IL-8, IL-8 (high sensitivity), IL-9, IP-10, MCP-1, MCP-4, MDC, MIP-1α, MIP-1β, MIP-3α, PIGF, SAA, TARC, Tie-2, TNF-α, TNF-β, TSLP, VCAM-1, VEGF-A, VEGF-C, VEGF-D, and VEGFR-1/Flt-1. Assays were run on a MESO QuickPlex SQ 120. Briefly, samples were incubated with the detection reagents at 25 °C for 2 h, followed by a wash step to remove unbound substances. The plate was then incubated with the readout reagent at room temperature for 30 min, after which the signal was measured. All incubations were performed on a shaker to ensure proper mixing. All analyte concentrations were calculated using Discovery Workbench software (version 4.0). Secretion data for all factors were normalized appropriately to protein content using the BCA assay (Pierce) as per manufacturer’s instructions, with no modifications made to the standard protocol.

### 2.10. Seahorse Analysis of Metabolic Profiles from OAC Tumour Explants and Generation of Tissue-Conditioned Media (TCM)

Fresh resected oesophageal adenocarcinoma tissue was collected from three patients at the time of oesophagectomy and processed within 30 min. All the oesophageal adenocarcinoma patients were males, with an average age of 69.6 ± 5.5 years and a BMI of 27.1 ± 5.5 kg/m^2^, who exhibited a poor response to neo-adjuvant therapy, as evidenced by a Tumour Regression Grade of 4 or 5. Each sample was divided into two portions to generate explants for treatment, with one receiving the vehicle control and the other being treated with AuNP-P3. Due to the limited size of the endoscopic biopsies obtained from patients, the samples could only be split into two, restricting our analysis to these two conditions (control and AuNP-P3). The timepoints for explants treatment and analysis were previously optimized [14]. Explants were cultured in 1 mL of M199 (Gibco, Thermofisher, Waltham, MA, USA) supplemented with 0.1% gentamicin (Lonza, Switzerland), in a 24-well plate (Sarstedt, Nümbrecht, Germany) for 2 h at 37 °C and 5% carbon dioxide in a humidified incubator (Thermofisher, Waltham, MA, USA) to allow for tissue equilibration. After this time, fresh media containing the treatment were added to the tissue [10 μM AuNP-P3, or control (0.1% water)]. After 18 h of culturing, the explants were subjected to Seahorse analysis and TCM was collected and stored. Fresh media were added, and the samples were subjected to 2 Gy irradiation and cultured for 6 h. After this time, the irradiated tissues were subjected to Seahorse analysis and TCM was collected and stored at −80 °C, as previously described [26]. Seahorse data were normalized to the total protein content using a BCA assay (Pierce).

### 2.11. Multiplex ELISA of Tissue Explants

The tissue-conditioned media (TCM) obtained in 2.7 were processed according to the MSD (Meso Scale Discovery, Rockville, MD, USA) multiplex protocol. To assess angiogenic, vascular injury, pro-inflammatory, and cytokine and chemokine secretions from ACM, a 54-plex ELISA kit separated across 7 plates was used (Meso Scale Discovery, Rockville, MD, USA). The TCM were run undiluted for all assays except Vascular Injury Panel 2, where a one-in-four dilution was used, as per previous optimization experiments. Secretion data for all factors were normalized appropriately to the protein content using the BCA assay (Pierce).

### 2.12. Zebrafish Husbandry, Maximum Tolerated Dose (MTD), and Intersegmental Vessel Assay

Tg(fli1:EGFP) zebrafish embryos (Danio rerio) were maintained in a recirculating aquaculture system, according to standard procedures and as previously described [19]. The Tg(fli1:EGFP) zebrafish model is widely used to study vascular development, particularly intersegmental vessel formation, due to its ability to label endothelial cells with green fluorescent protein, allowing for the real-time visualization of blood vessel growth. At 6 h post-fertilization (hpf) Tg(fli1:EGFP), viable zebrafish embryos were collected and plated at a rate of 5 per well in a 48-well plate, using an Olympus-SZX16 microscope (Olympus corporation, Tokyo, Japan) in a 500 μL embryo medium (5 mM NaCl, 0.17 mM KCl, 0.4 mM CaCl_2_ and 0.16 mM MgSO_4_). The embryo medium was removed, and a 500 μL-containing embryo medium and control or test compound were added to specific wells. Sunitinib, a receptor tyrosine kinase inhibitor with potent antiangiogenic activity, was used as a positive control for the assay. P3, AuNPs, AuNP-P3, and sunitinib were used at a concentration of 10 μM for experiments. The control was 0.1% DMSO + 0.1% water in embryo media. To determine the MTD of AuNPs and AuNP-P3 administered in embryo media, a dilution series was prepared (10 μM to 50 μM) and compared to the control. The embryos were maintained at 28 °C, and survival was recorded 2 days post-fertilization (dpf). After viability analysis, larvae were dechorionated under an Olympus-SZX16 microscope, euthanized, and fixed in 400 μL of 4% paraformaldehyde. An intersegmental vessel assay (ISV) and the quantification of intersegmental vessels were performed as previously described [19]. Experiments were repeated 3 times with a total of 15 embryos per condition.

### 2.13. Statistical Analysis

Statistical analysis was performed using GraphPad Prism version 10.0.2 software (GraphPad Software, San Diego, CA, USA). Scientific data were expressed as mean ± standard error of the mean (SEM). *T*-tests were employed for comparisons between two groups, with the choice between paired and unpaired *t*-tests determined by the relationship between the data sets. Paired *t*-tests were applied for related or matched groups (e.g., the same cell line before and after treatment), while unpaired *t*-tests were used for independent groups (e.g., different subjects or cell lines). For comparisons involving multiple groups, ANOVA was utilized to assess the differences in means across more than two groups, with Šídák’s or Tukey’s correction applied to control the risk of Type I error in multiple comparisons. The specific statistical tests used are indicated in figure legends. For all statistical analyses, differences were considered statistically significant at *p* < 0.05. For the correlation analysis between the 54-plex ELISA data and Seahorse data, Spearman correlations were carried out using Graphpad 10.0.2 software [24]. Correlation analysis was performed with this approach due to the sample size available and the heterogeneous distribution of data, which arises from the varying upper and lower limits of detection across the 54 analytes in the MSD V-PLEX assay. Spearman correlations are robust in their consideration of monotonic relationships whilst still accounting for potential outliers associated with high-dimensional data measurements. Graphical representations of correlations were generated with R software version 4.1.1 and R package ‘corrplot’ version 0.92 [26]. All correlations with an associated *p*-value < 0.05 were considered statistically significant. The Holm–Bonferroni post hoc correction was used to control for multiple comparison testing during correlation analysis.

## 3. Results

### 3.1. Pyrazinib-Functionalised Gold Nanoparticles (AuNP-P3) Significantly Increased Radiosensitivity In Vitro in an Isogenic Model of Radioresistant Oesophageal Adenocarcinoma

We have previously shown that 10 μM pyrazinib (P3) for 24 h prior to radiation is the optimal dose to enhance radiosensitivity in both radiation-sensitive OE33P and radioresistant OE33R cells [19].

We generated a new combination compound, coupling P3 to PEGylated gold nanoparticle (PEG-AuNPs, also labelled as “AuNPs” for convenience in the manuscript). The chemical functionalization of P3 was carried out through electrostatic charges between the carboxylic chemical groups onto PEG-AuNPs and the N-H group under basic conditions. In Supplementary Figure S1, we observe a red shift of the plasmon peak from 520 nm (PEG-AuNPs) (Figure S1A,B black line) to 540 nm after the P3 conjugation by electrostatic adsorption (AuNP-P3) (Figure S1A,B red line). The representative TEM image of PEG-AuNPs shows spheric gold nanoparticles, decorated by a layer of PEG with a nominal particle size of approximately 4.6 ± 1.0 nm (Supplementary Figure S1C). Similarly, the representative TEM image of AuNP-P3 shows larger particles with approximately a nominal diameter of 8.9 ± 1.6 nm (Supplementary Figure S1D). Interestingly, the hydrodynamic size was confirmed by DLS and NTA measurements, as reported in Supplementary Figure S1E–H. Particle size results are in agreement between DLS and NTA measurements for both the hydrodynamic diameter and dispersion homogeneity, as summarized in Supplementary Table S1, and are in line with the proposed particle size distribution orthogonal approach [27]. Furthermore, NTA provided details on the particle concentration values for the two AuNPs, as from the details presented in Supplementary Figure S1G,H. As shown in Supplementary Figure S1I,L, zeta potential measurements show that all AuNPs were colloidally stable at a physiological pH of 7.4. AuNP-P3 charge was more positive than PEG-AuNPs as controls, confirming the complete neutralization of the negative charge of PEG diacide during the coupling with the P3. Moreover, the UV-Vis spectra of AuNP-P3 remain unaltered after storage at room temperature, confirming the formation of stable colloidal solution (Supplementary Figure S1A,B). A summary of gold nanoparticle characterization is reported in Supplementary Table S1.

**Figure 1:**
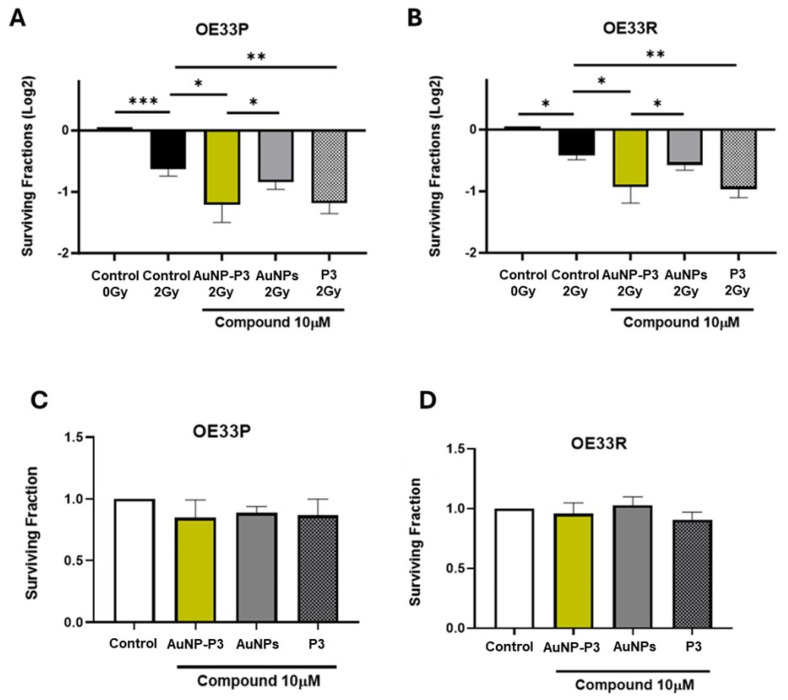
Pyrazinib-functionalised gold nanoparticles (AuNP-P3) enhanced radiosensitivity in an the OAC cell line model of radioresistance. The effect of pyrazinib-coupled gold nanoparticles (AuNP-P3), pyrazinib (P3), and gold nanoparticles (AuNPs) at 10 µM treatment on the radiosensitivity of OE33P (radiation sensitive) and OE33R (radioresistant) cells was assessed by clonogenic assays and compared to the control (0.1% DMSO + 0.1% water). Surviving fraction of OE33P (**A**) and OE33R (**B**) cells following treatment with compounds at 10 µM and 2 Gy X-ray radiation. Clonogenic assay was used to assess cell survival based on the ability of a single cell to grow into a colony after receiving treatment with the compound at 10 µM. Cell viability was assessed in mock-radiated OE33P (**C**) and OE33R cells (**D**). Data are expressed as mean ± SEM. Statistical analysis was carried out using a two-tailed paired *t*-test. * *p* < 0.05, ** *p* < 0.01, *** *p* < 0.001 (n = 4).

The ability of AuNP-P3 to increase radiosensitivity in OAC was evaluated using a clonogenic assay in OE33P and OE33R cells exposed to 2 Gy X-ray radiation. Treatment with 10 μM AuNP-P3 significantly enhanced radiosensitivity in both OE33P and OE33R cells compared to the vehicle control (*p* = 0.0005, *p* = 0.0001, respectively) and to 10 μM unloaded gold nanoparticles (AuNPs) (*p* = 0.0481, *p* = 0.0381, respectively). Additionally, 10 μM P3 enhanced radiosensitivity following 2 Gy irradiation in both OE33P (*p* = 0.0005) and OE33R cells (*p* = 0.0001) (Figure 1A,B). Notably, the radiosensitizing effect of AuNP-P3 was not associated with decreased cell viability, as assessed by clonogenic assay in mock-irradiated OE33P (Figure 1C) and OE33R cells (Figure 1D), with no significant change in viability detected at 10 μM treatment with AuNP-P3, P3, or AuNPs.

### 3.2. Pyrazinib-Functionalised Gold Nanoparticles (AuNP-P3) Significantly Altered Metabolic Rates in an Isogenic Model of Radioresistant Oesophageal Adenocarcinoma

**Figure 2:**
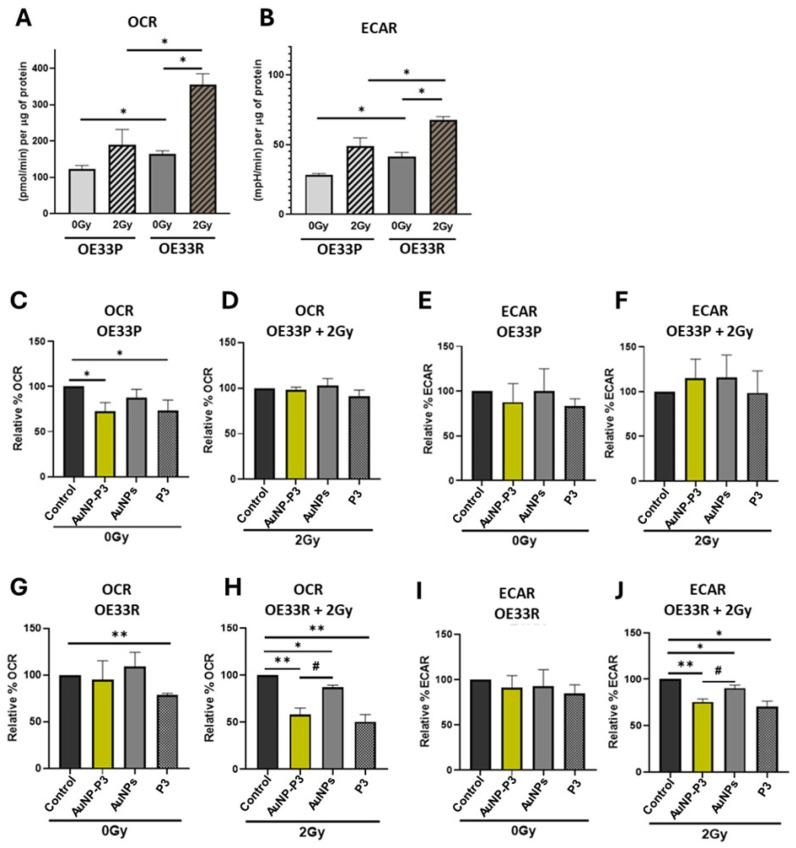
Pyrazinib-functionalised gold nanoparticles (AuNP-P3) treatment reduced oxidative phosphorylation and glycolysis in response to ionizing radiation in an OAC cell line model of radioresistance. OE33P (radiation sensitive cells) and OE33R (radiation resistant cells) were treated for 24 h with the compounds at 10 µM or with the control (0.1% DMSO + 0.1% water) and subsequently irradiated at 2 Gy X-ray radiation. After 24 h, cells were used to measure metabolic rates and the supernatants were stored for multiplex ELISA. Oxygen consumption rate (OCR) and extracellular acidification rate (ECAR) were measured in real-time using Seahorse Biosciences XFe24 analyser. Comparison of basal and radiation-induced OCR (**A**) and ECAR (**B**) levels between OE33P and OE33R cells. Relative percentage OCR (**C**,**D**) and ECAR (**E**,**F**) in OE33P cells following treatment with compounds at 10 μM and mock or 2 Gy radiation. Relative percentage OCR (**G**,**H**) and ECAR (**I**,**J**) in OE33R cells following treatment with compounds at 10 μM and mock or 2 Gy radiation. Data are expressed as mean ± SEM. Statistical analysis was carried out using an unpaired *t*-test to compare different cell lines and paired *t*-test to compare within the same cell line. * *p* < 0.05, ** *p* < 0.01 (Compound vs. Control); # *p* < 0.05 (AuNP-P3 vs. AuNPs) (n = 3).

We have previously demonstrated that pyrazinib (P3) significantly reduced measures of mitochondrial metabolism in OAC cells [19]. To determine if the conjugated form of pyrazinib with gold nanoparticles (AuNP-P3) maintains a similar bioactivity, we used an isogenic OAC cell line model of radioresistance: OE33P (radiation-sensitive) and OE33R (radioresistant) cells. The effect of AuNP-P3 on mitochondrial metabolism was assessed using the Seahorse XFe24 analyser. OCR (oxygen consumption rate) and ECAR (extracellular acidification rate) were used to assess cellular metabolism. OCR reflects the rate at which cells consume oxygen, indicating mitochondrial respiration and energy production through oxidative phosphorylation. In contrast, ECAR measures the acidification of the surrounding medium during glycolysis, providing insight into glycolytic activity. Radioresistant OE33R cells showed a significantly higher oxygen consumption rate (OCR), indicating increased oxidative phosphorylation (Figure 2A), and extracellular acidification rate (ECAR), indicating higher glycolysis (Figure 2B), at baseline compared to OE33P cells (*p* = 0.0286 and *p* = 0.02, respectively). Similarly, under 2 Gy radiation, OE33R cells exhibited a significantly elevated OCR and ECAR compared to OE33P cells (*p* = 0.0338 and *p* = 0.0437, respectively) (Figure 2A,B). Notably, only the radioresistant OE33R cells showed a significant increase in OCR and ECAR levels under 2 Gy radiation compared to OE33R cells without radiation (0 Gy) (*p* = 0.0234 and *p* = 0.021, respectively) (Figure 2A,B). A 24 h treatment with 10µM AuNP-P3 and P3 significantly reduced OCR levels in OE33P cells without radiation (*p* = 0.0396 and *p* = 0.0167, respectively) (Figure 2C), an effect not observed under 2 Gy radiation (Figure 2D) or at the ECAR level (Figure 2E,F). No significant effect was detected with 10 µM unloaded gold nanoparticles (AuNPs). In contrast, in OE33R cells, 10 µM AuNP-P3 did not induce a significant change in OCR levels without radiation (Figure 2G), while 10 µM AuNP-P3 significantly reduced OCR levels under 2 Gy radiation (*p* = 0.027) (Figure 2H). Furthermore, 10µM P3 significantly reduced OCR levels in OE33R cells both without and with 2 Gy radiation (*p* = 0.0024 and *p* = 0.0067, respectively). Additionally, 10 µM AuNPs significantly decreased the OCR in OE33R cells subjected to 2 Gy radiation (*p* = 0.0114), an effect not observed in non-irradiated cells. Interestingly, AuNP-P3 significantly reduced OCR levels compared to AuNPs in irradiated OE33R cells (*p* = 0.0089) (Figure 2G,H). Similar effects were observed with ECAR in OE33R cells following 2 Gy radiation: 10 µM AuNP-P3, P3, and AuNPs significantly decreased ECAR levels compared to the control (*p* = 0.005, *p* = 0.0133, *p* = 0.0345, respectively), and AuNP-P3 significantly reduced ECAR levels compared to AuNPs in irradiated OE33R cells (*p* = 0.0415) (Figure 2I,J). Furthermore, similar results were obtained with the analysis of other mitochondrial parameters such as ATP turnover, proton leak, maximal respiration, and non-mitochondrial oxygen consumption (Supplementary Figure S2). No differences were seen in the coupling efficiency and spare respiratory capacity with 10µM AuNP-P3, P3, and AuNPs (Supplementary Figure S2).

This screen demonstrated that AuNP-P3 significantly reduces the OCR and ECAR in OE33R cells after 2 Gy irradiation, highlighting its potential as an effective radiosensitizer with antimetabolic activity in the neo-adjuvant setting.

### 3.3. Pyrazinib-Functionalised Gold Nanoparticles (AuNP-P3) Significantly Altered the Levels of Secreted Mediators in Response to Ionizing Radiation in an Isogenic Model of Radioresistant Oesophageal Adenocarcinoma

**Figure 3:**
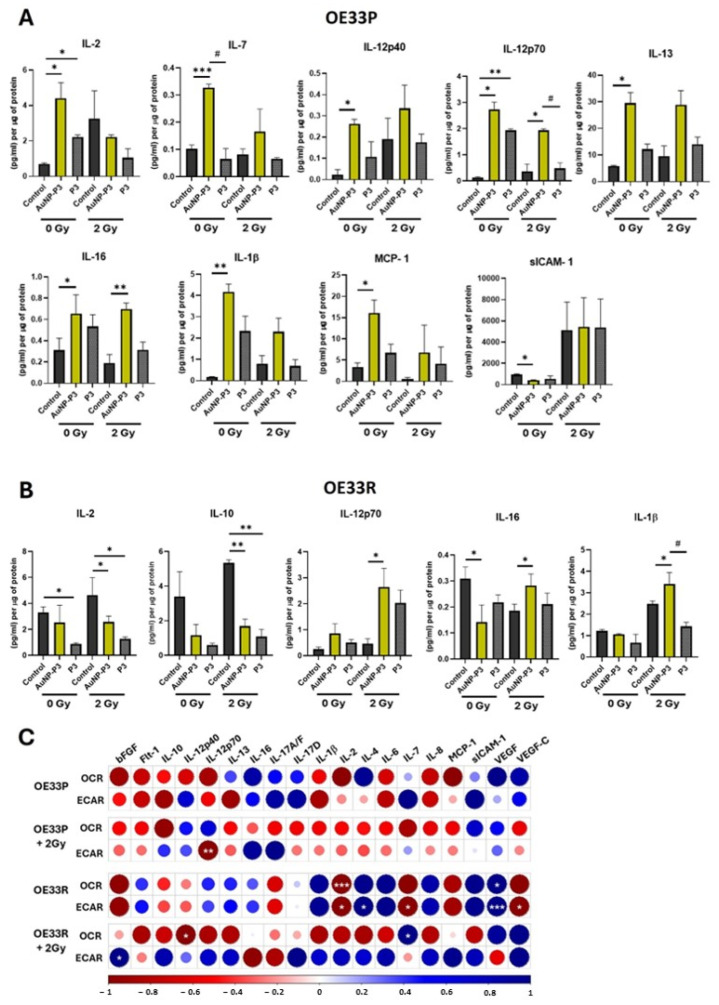
Pyrazinib-functionalised gold nanoparticles (AuNP-P3) significantly altered the levels of secreted mediators in response to ionizing radiation in an OAC cell line model of radioresistance. The secreted levels of protein in OE33P (radiation sensitive) and OE33R (radiation resistant) cells were evaluated by 54-plex ELISA. Here, we report data only for the significantly modulated proteins. (**A**) AuNP-P3 10 µM significantly alters the level of 9 proteins (IL-2, IL-7, IL-12p40, IL12-p70, IL-13, IL-16, IL1β, MCP-1, and sICAM-1) in OE33P cells (radiosensitive) when compared to the control (0.1% DMSO). (**B**) AuNP-P3 10 µM significantly alters the level of 5 proteins (IL-2, IL-10, IL12-p70, IL-16, and IL1β) in OE33R cells (radioresistant) when compared to the control (0.1% DMSO + 0.1% water). Two-tailed paired *t*-test; * *p* < 0.05, ** *p* < 0.01, *** *p* < 0.001, # *p* < 0.05 (P3 vs. AuNP-P3) (n = 3). Data are expressed as mean ± SEM (n = 3). (**C**) Correlation plots showing significant correlations between the levels of secreted proteins and the metabolic rates in OE33P and OE33R cells treated with AuNP-P3 10 µM in the presence or absence of 2 Gy radiation. (Spearman correlation, blue indicates positive correlations and red indicates inverse negative correlations). The Holm–Bonferroni hoc correction was used to control for multiple comparison testing. * *p* < 0.05, ** *p* < 0.01, *** *p* < 0.001.

We investigated the effects of AuNP-P3 on the modulation of secreted proteins in OE33P (radiosensitive) and OE33R (radioresistant) cells, both in the presence and absence of 2 Gy radiation. In mock-irradiated OE33P cells, 10 μM AuNP-P3 significantly increased the levels of IL-2 (*p* = 0.0136), IL-7 (*p* < 0.0001), IL-12p40 (*p* = 0.0297), IL-12p70 (*p* = 0.0132), IL-13 (*p* = 0.0270), IL-16 (*p* = 0.0319), IL-1β (*p* = 0.0076), and MCP-1 (*p* = 0.0165) while decreasing the level of sICAM-1 (*p* = 0.0051) (Figure 3A). This effect was lost in the presence of 2 Gy radiation, except for IL-12p70 and IL-16, which were still significantly increased following treatment with 10 μM AuNP-P3 (*p* = 0.025 and *p* = 0.0052, respectively) (Figure 3A). Then, 10 μM P3 significantly increased the levels of IL-12p70 and IL-2 (*p* = 0.0011 and *p* = 0.0065, respectively) in the absence of 2 Gy radiation.

In radioresistant OE33R cells, 10 μM AuNP-P3 significantly decreased the levels of IL-2 (*p* = 0.0049) and IL-16 (*p* = 0.0173) in mock-irradiated cells (Figure 3B). Following 2 Gy radiation, AuNP-P3 significantly reduced the levels of IL-2 (*p* = 0.0483) and IL-10 (*p* = 0.0095), while significantly increasing the levels of IL-12p70 (*p* = 0.0399), IL-16 (*p* = 0.04), and IL-1β (*p* = 0.035) (Figure 3B). Furthermore, 10 μM P3 significantly increased the levels of IL-2 and IL-10 (*p* = 0.038 and *p* = 0.002, respectively) in the presence of 2 Gy radiation.

Strong correlations were observed between the levels of mediators released by OE33P and OE33R cells treated with 10 μM AuNP-P3 and their metabolic parameters (OCR and ECAR) (Figure 3C). No significant correlations were observed in AuNP-P3-treated OE33P cells at 0 Gy radiation, whereas a significant negative correlation was detected between ECAR and IL-12p70 levels in 2 Gy-irradiated OE33P cells (Figure 3C). In radioresistant OE33R cells, significant correlations were observed in AuNP-P3-treated mock-irradiated cells between the OCR and IL-2 and VEGF and between the ECAR and IL-2, IL-7, VEGF, and VEGF-C (Figure 3C). Additionally, significant correlations were measured in AuNP-P3-treated 2 Gy-irradiated OE33R cells between the OCR and IL-12p40 and IL-7 and between the ECAR and bFGF (Figure 3C). Furthermore, significant correlations were found between protein secretions and other mitochondrial metabolic parameters, including proton leak, maximal respiration, and ATP-linked respiration, in both cell lines before and after 2 Gy radiation (Supplementary Figure S3). Further investigating the mechanisms underlying the observed changes in secreted proteins could enhance the potential of AuNP-P3 as a therapeutic agent. Understanding how AuNP-P3 modulates protein secretion can reveal key pathways involved in immune activation, tumour response, and radioresistance, ultimately improving its efficacy and targeting in cancer treatment.

### 3.4. Pyrazinib-Functionalised Gold Nanoparticles (AuNP-P3) Altered Metabolic Parameters and Secretome of OAC Tumour Explants in Response to Ionizing Radiation

**Figure 4:**
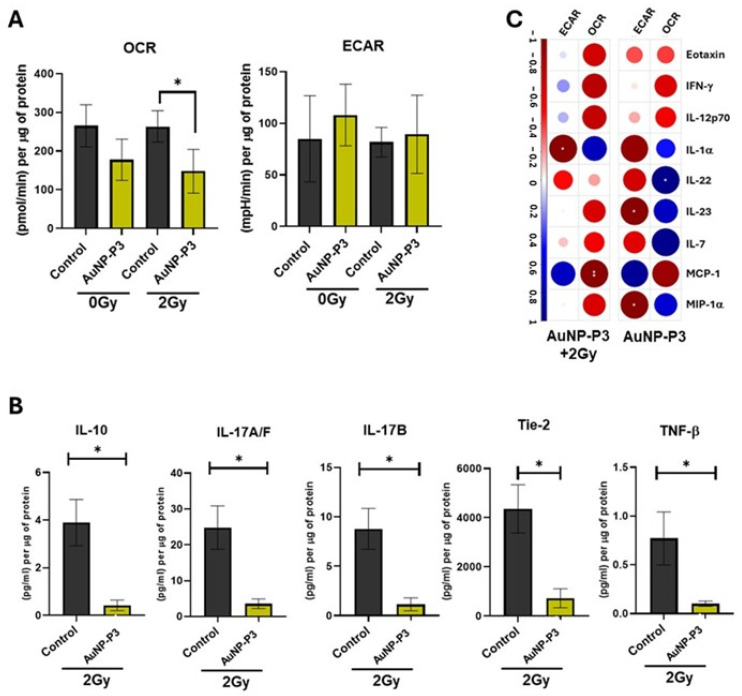
Pyrazinib-functionalised gold nanoparticles (AuNP-P3) decreased metabolic parameters and released mediators in OAC tumour explants in response to ionizing radiation. OAC explants were treated with pyrazinib-functionalised gold nanoparticles (AuNP-P3) at 10 µM, and after 18 h incubation, explants were exposed to 2 Gy X-ray radiation. After 6h incubation, OCR and ECAR were measured in real-time. Tissue-conditioned media were collected after Seahorse measurement and used for multiplex ELISA analysis. (**A**) AuNP-P3 significantly inhibits OCR in OAC explants exposed to 2 Gy radiation, when compared to the control (0.1% water). ANOVA with Šídák’s multiple comparisons test, * *p*  <  0.05. Data expressed as mean  ±  SEM (n = 3). (**B**) Altered released cytokines in AuNP-P3 treated OAC explants subjected to 2 Gy X-ray radiation were measured by multiplex ELISA. Two-tailed unpaired *t*-test; * *p* < 0.5. Data expressed as mean  ±  SEM (n = 3). (**C**) Correlation plots showing significant correlations between the levels of secreted proteins and the metabolic rates in huma OAC fresh explant treated with AuNP-P3 10µM in the presence or absence of 2 Gy radiation. Spearman correlation; blue indicates positive correlations and red indicates inverse negative correlations. The Holm–Bonferroni hoc correction was used to control for multiple comparison testing. * *p* < 0.05, ** *p* < 0.01.

We investigated the effects of 10 μM AuNP-P3 on metabolic rates and secretome in fresh OAC tumour tissue obtained during oesophagectomy. We measured the oxygen consumption rate (OCR) and extracellular acidification rate (ECAR) in AuNP-P3-treated explants in the presence and absence of 2 Gy X-ray irradiation. Tissue-conditioned media were analyzed using multiplex ELISA. AuNP-P3 significantly reduced OCR levels in explants subjected to 2 Gy radiation compared to control (*p* = 0.0484) (Figure 4A). No significant changes were measured in the absence of radiation. No significant changes were measured in ECAR levels (Figure 4A). The significant decreased secretion of proteins was observed following 10 μM AuNP-P3 treatment in the presence of ionizing radiation, including IL-10, IL-17A/F, IL-17B, Tie-2, and TNF-β (Figure 4B). Strong correlations were noted between released proteins levels and mitochondrial metabolic parameters (OCR and ECAR) in OAC tissue explants treated with 10 μM AuNP-P3, both in the absence and presence of 2 Gy X-ray radiation. Notably, significant positive correlations were observed between IL-22 and the OCR in the absence of radiation, while significant negative correlations were detected between the ECAR and IL-23 and MIP-1α. In the presence of 2 Gy X-ray radiation, significant negative correlations were observed between MCP-1 and the OCR and between the ECAR and IL-1α in AuNP-P3-treated explants (Figure 4C). These findings demonstrate the bioactivity of AuNP-P3 in modulating the secretion of pro-inflammatory and angiogenic mediators in response to ionizing radiation in OE33 radiosensitive and radioresistant cells.

### 3.5. Pyrazinib-Functionalised Gold Nanoparticles (AuNP-P3) Significantly Inhibited Developmental Angiogenesis In Vivo in Tg(fli1:EGFP) Zebrafish

**Figure 5:**
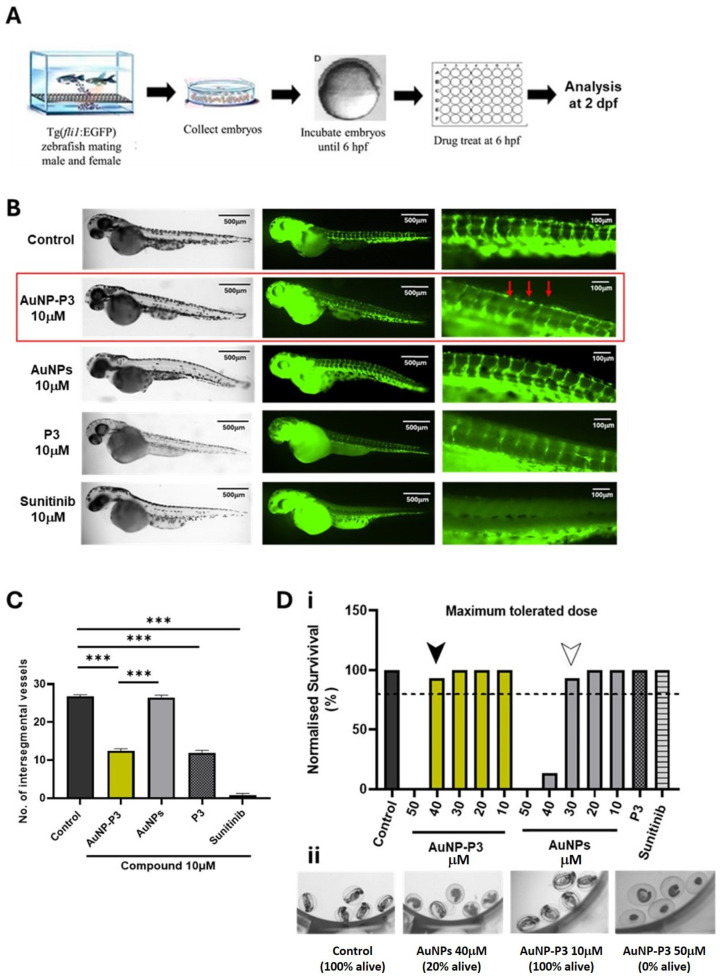
Pyrazinib-functionalised gold nanoparticles (AuNP-P3) inhibited developmental angiogenesis in Tg(fli1:EGFP) zebrafish. (**A**) Intersegmental vessel assay schematic; 6 h post-fertilization (hpf) Tg(fli1) embryos were treated with compounds and fixed and analyzed at 2 days post-fertilization (dpf). (**B**) Representative fluorescent images illustrating GFP-positive intersegmental vessels at low and high magnification and brightfield images of whole larvae at 2 dpf in Tg(fli1) zebrafish. Then, 6 hpf embryos were treated with the control (0.1% DMSO) C, pyrazinib-functionalised gold nanoparticles (AuNP-P3) 10 μM, AuNPs (gold nanoparticles) 10 μM, pyrazinib (P3) 10 μM, and sunitinib 10 μM as indicated on the graph. Red arrows indicate the altered vessel formation observed with AuNP-P3 treatment. (**C**) The number of intersegmental vessels was quantified at 2 dpf. One-way ANOVA with Tukey’s multiple comparisons test. Data are expressed as mean ± SEM; *** *p* < 0.001 (n = 15). (**D**) (**i**) Maximum tolerated dose (MTD) of the compounds in the Tg(fli1) embryos. Then, 6 hpf embryos were treated with 0.1% DMSO control, AuNP-P3 10, 20, 30, 40, and 50 μM, AuNPs (gold nanoparticles) 10, 20, 30, 40, and 50 μM, pyrazinib (P3) 10 μM, and sunitinib 10 μM. The dotted line denotes the 80% survival rate used as a cut-off for the establishment of the MTD. The survival of zebrafish treated with AuNP-P3 exceeded 80% at concentrations ≤ 40 μM (black arrow), while at concentrations ≤ 30 μM (white arrow) for AuNPs (n = 15). (**ii**) Representative images of live and dead zebrafish larvae at 2 dpf.

We previously demonstrated that 10 μM pyrazinib (P3) exhibits potent anti-angiogenic activity by inhibiting blood vessel development in zebrafish embryos. In this study, using Tg(fli1) embryos, we screened pyrazinib-loaded gold nanoparticles (AuNP-P3), unloaded gold nanoparticles (AuNPs), and P3 for anti-angiogenic activity with a phenotype-based approach employing the intersegmental vessel (ISV) assay, as illustrated in Figure 5A. Sunitinib, a receptor tyrosine kinase inhibitor known for its potent anti-angiogenic activity, served as a positive control.

The maximum tolerated dose of AuNP-P3 and AuNPs was determined at 48 h post-fertilization (hpf), revealing that embryo survival exceeded 80% at concentrations below 40 μM for AuNP-P3 and 30 μM for AuNPs (Figure 5D(i,ii)). P3 and sunitinib did not affect survival at 10 μM.

All compounds were tested at 10 μM for their anti-angiogenic activity by quantifying ISV numbers at 48 hpf. The results showed that 10 μM AuNP-P3 significantly reduced ISV numbers by approximately 54% (*p* < 0.0001), while 10 μM P3 reduced ISV numbers by approximately 58% (*p* < 0.0001). Sunitinib at 10 μM showed the most significant reduction in ISV numbers, approximately 96% (*p* < 0.0001) (Figure 5C). Interestingly, 10 μM AuNPs did not exhibit anti-angiogenic effects compared to the control. However, a significant effect was observed when comparing 10 μM AuNPs to 10 μM AuNP-P3 (*p* < 0.0001), indicating that the anti-angiogenic effect of AuNP-P3 is mediated by P3 and is independent of the gold nanoparticles themselves.

Fluorescent images (Figure 5B) illustrated the potent reduction in ISV numbers and the loss of vessel integrity following treatment with 10 μM AuNP-P3. No gross morphological defects or toxicities were observed following treatment with 10 μM AuNP-P3, AuNPs, P3, or sunitinib, although there were some alterations in skin pigmentation following P3 treatment (Figure 5B).

## 4. Discussion

The primary objective of this study was to develop a gold nanoparticle formulation of P3 (AuNP-P3) that maintains the radio-sensitizing properties of P3 while addressing its limited solubility. Specifically, while P3 is readily soluble in DMSO, its poor solubility in water-based solutions limits its direct application in biological systems in vivo. By conjugating P3 to pegylated AuNPs, we successfully formulated AuNP-P3, which is highly soluble in aqueous environments. This enhancement is consistent with the well-documented ability of gold nanoparticles to improve the solubility of hydrophobic compounds by optimizing surface coverage and leveraging increased dynamic forces during functionalization [23,28]. This improvement not only preserves P3’s bioactivity but also provides a robust platform for its potential targeted delivery in the treatment of OAC [29]. The integration of in vitro, ex vivo, and in vivo models in this study provides a robust framework for understanding the multifaceted effects of AuNP-P3 on oesophageal adenocarcinoma OAC. Each model offers unique contributions to the overall interpretation of the findings. In vitro studies were pivotal for elucidating the cellular mechanisms of AuNP-P3, particularly its effects on radiosensitivity, metabolic modulation, and cytokine secretion in both radiosensitive and radioresistant OAC cells. Ex vivo tumour explants complemented these findings by validating the compound’s anti-inflammatory and anti-metabolic effects in a more physiologically relevant tumour microenvironment, bridging the gap between cellular assays and clinical conditions. Meanwhile, the in vivo zebrafish model uniquely demonstrated AuNP-P3’s ability to inhibit angiogenesis in a biologically intact system, emphasizing its potential impact on tumour vascularization and progression. We demonstrated that AuNP-P3 is soluble in water and bioactive. Our findings revealed that AuNP-P3 significantly enhanced radiosensitivity in both radiation-sensitive and radiation-resistant OAC cells at a similar level to P3. Notably, there were no significant differences observed when comparing P3 with AuNP-P3. Although unconjugated AuNPs exhibited a trend towards decreasing the surviving fraction in both radiosensitive and radioresistant cells, the effect was not statistically significant (Figure 1A,B). These results suggest that the radiosensitizing effect of AuNP-P3 is mediated by P3, and no additive effects were detected with gold nanoparticles. Furthermore, the radiosensitizing effect of AuNP-P3 was independent of changes in cellular proliferation or cytotoxicity (Figure 1C,D). Importantly, while AuNP-P3 did not demonstrate superiority in enhancing the radiosensitizing effect of P3, the conjugated formulation remained active. Crucially, P3 did not alter its bioactivity when combined with this formulation of gold nanoparticles. This suggests that AuNP-P3 holds promise as a potential therapeutic agent for enhancing radiosensitivity in oesophageal adenocarcinoma, offering a novel approach for targeted drug delivery in neoadjuvant radiochemotherapy. Furthermore, 2 Gy radiation was selected based on the fact that it is the most clinically relevant dose for oesophageal cancer patients undergoing radiation therapy. This aligns with clinical treatment regimens, where 2 Gy is a common fractionation dose [30]. Importantly, in our previous work on P3 in oesophageal cancer [19], we observed sustained radiosensitivity at higher doses of X-ray radiation (4 and 6 Gy), but the most significant and reproducible effects were consistently seen at 2 Gy, particularly in terms of statistical significance. Therefore, the ability to enhance radiosensitivity at this dose makes it a meaningful and practical focus for therapeutic strategies, as it mirrors the typical radiation dose used in clinical settings. By using 2 Gy, we ensure the relevance of our findings to real-world applications in oesophageal cancer therapy and increase the potential for translation into clinical practice.

Dysregulated cellular energetics emerges as a hallmark of cancer and treatment resistance [27]. Cancer cells modify their metabolism to sustain rapid growth and proliferation, with recent research associating immune-metabolic profiles with key oncologic factors such as treatment resistance and increased invasiveness [8,31,32]. Ionizing radiation prompts cancer cells to predominantly utilize oxidative phosphorylation to cope with genotoxic stress [33] and induce mitochondrial biogenesis [34]. Pyrazinib (P3) significantly impacts the oxidative phosphorylation rate in OAC tumours, potentially exerting an anti-cancer effect and enhancing radioresponse, as demonstrated in vitro in an isogenic model of OAC radioresistance [19]. In our study, 2 Gy radiation markedly increased oxidative phosphorylation and glycolysis in radioresistant cells. AuNP-P3 notably decreased both OCR and ECAR in radiated cells, indicating the inhibition of both pathways (Figure 2). These effects were absent in radiosensitive cells, suggesting that AuNP-P3 acts downstream to regulate both pathways, unaffected by radiation-induced metabolic upregulation.

Moreover, AuNP-P3 significantly reduced oxidative phosphorylation in ex vivo tumour explants post-radiation (Figure 4). Targeting oxidative phosphorylation has proven successful in enhancing radiosensitivity and reducing tumour growth [35,36]. Considering the prevalence of oxidative phosphorylation in OAC tumours and AuNP-P3’s ability to reduce it in the presence of radiation, the further development of AuNP-P3 as a novel radiosensitizer in OAC is warranted. One limitation is the small sample size of fresh human explants, all from male patients. Future studies should include a larger patient cohort to address potential gender-related treatment response biases.

Cellular metabolism is intricately intersected with local and systemic inflammation [37]. OAC is an inflammation-driven cancer [38], with inflammation-altered metabolism associated with treatment response [26]. Previously, we demonstrated the elevated secretion of inflammatory and angiogenic factors in OE33R radioresistant cells compared to OE33P cells, implicating these mediators in OAC radioresistance [19]. Pyrazinib (P3) notably reduced secretions of numerous mediators in OE33R cells [19]. We investigated AuNP-P3’s effects on inflammatory profiles in vitro and ex vivo, with and without ionizing radiation. In radiosensitive cells, AuNP-P3 significantly modulated secreted factors including ICAM-1, MCP-1, IL-2, IL-7, IL-12p40, IL-12p70, IL-13, IL-16, and IL-1b (Figure 3A). Following 2 Gy radiation, these effects diminished, except for IL-12p70 and IL-16. In radioresistant cells, AuNP-P3 significantly affected IL-2, IL-10, IL-16, IL-12p70, and IL-1b post-radiation (Figure 3B). The data show that most of these molecules exhibit similar trends between P3 and AuNP-P3 in terms of their effects on protein expression. When AuNP-P3 alters the expression of a given molecule, P3 often shows a similar trend, although not always reaching statistical significance. This suggests that the presence of AuNPs does not drastically alter the biological activity of P3 in terms of protein secretion for most markers. The exceptions to this trend are IL-7 and IL-1β, where a significant increase in expression is observed with AuNP-P3, but P3 alone does not show the same trend. This discrepancy could be due to differences in how the AuNP-P3 formulation interacts with the cell system, potentially enhancing the release or activation of these cytokines. Despite these minor differences, the overall conclusion is that AuNP-P3 maintains similar bioactivities to P3, which is consistent with its overall therapeutic potential across the various experimental models reported in this work. In particular, the observed alterations in cytokine profiles following treatment with AuNP-P3 offer important insights into the immune mechanisms involved. Notably, the significant upregulation of IL-12p70 in both irradiated and non-irradiated cell lines suggests an enhancement of anti-tumour immunity. IL-12p70 is crucial for promoting Th1 responses, which activate cytotoxic T cells and NK cells, key players in effective anti-tumour immunity [39]. This upregulation indicates that AuNP-P3 may help shift the immune environment towards a Th1-dominated response, potentially improving tumour control and increasing radiation sensitivity. Similarly, the upregulation of IL-16, a chemotactic cytokine [40], could attract immune cells to the tumour microenvironment, potentially enhancing anti-tumour responses, and IL-1β can promote immune cell activation [41], which may contribute to the therapeutic effects of AuNP-P3. IL-2 is another cytokine known for its role in promoting T-cell proliferation and activation. It has been used in immunotherapy to enhance the immune response against tumours. The increased levels of IL-2 observed with AuNP-P3 could contribute to enhancing the cytotoxic immune response, potentially improving the efficacy of treatments like radiotherapy [42]. Similarly to IL-12p70, IL-12p40 is a key cytokine involved in promoting Th1 responses and activating cytotoxic T lymphocytes. The upregulation of IL-12p40 could enhance tumour cell recognition and destruction by cytotoxic T lymphocytes, increasing tumour radiosensitivity [43]. Furthermore, the increase in IL-7 with AuNP-P3 can enhance T-cell activity, improving tumour radiosensitivity by boosting immune responses against cancer cells [44]. MCP-1 is a Monocyte Chemoattractant Protein involved in the recruitment of monocytes to the site of inflammation. The elevated expression of MCP-1 with AuNP-P3 could potentially recruit pro-tumour macrophages to the tumour microenvironment, which may either support or hinder the efficacy of radiotherapy depending on the macrophage polarization [45]. Moreover, the decreased expression of ICAM-1 with AuNP-P3 could have a dual benefit by minimizing inflammatory responses that may otherwise support tumour growth and reduce the pro-metastatic properties of ICAM-1 [46]. Interestingly, IL-10 was significantly decreased in both radioresistant cell lines and in human explants treated with AuNP-P3. IL-10 has been identified as a key player in promoting radioresistance in cancer cells. Studies have shown that interleukin-10, along with other interleukins such as IL-1 and IL-4, can activate pathways like NF-κB, contributing to cancer radioresistance [47,48]; therefore, strategies to inhibit IL-10 or its signalling pathways could enhance the efficacy of radiation therapy. Therefore, the alteration of IL-10 in both our models reinforces the hypothesis that IL-10 could be a target for drug development and a robust marker of treatment response to AuNP-P3. These findings suggest that AuNP-P3 not only sensitizes tumours to radiation, but may also stimulate immune responses that support tumour eradication.

The impact of 2 Gy radiation on metabolic rates and the secretome warrants further exploration to understand how radiation interacts with AuNP-P3 treatment. Radiation induces stress that alters cellular metabolism, and these changes are modulated by AuNP-P3, potentially sensitizing cells to radiation by shifting metabolic pathways. Additionally, radiation triggers an inflammatory response that affects cytokine secretion in the tumour microenvironment. Understanding these interactions will provide insights into how AuNP-P3 could improve radiation therapy outcomes by enhancing immune responses and overcoming resistance.

The correlations observed between the mediators released by OE33P and OE33R cells treated with AuNP-P3 and their metabolic parameters (OCR and ECAR) highlight intriguing relationships between cellular metabolism and secretome profiles. In radioresistant OE33R cells, distinct patterns emerged under different treatment conditions. For instance, in mock-irradiated radioresistant cells, a positive correlation was observed between OCR and VEGF, as well as ECAR and VEGF, suggesting that VEGF secretion is closely tied to metabolic activity, potentially supporting angiogenesis and tumour growth. In 2 Gy-irradiated OE33R cells, a positive correlation between ECAR and bFGF was detected, indicating a link between increased glycolytic activity and bFGF secretion, which is known to promote angiogenesis and tumour progression. Meanwhile, negative correlations were observed between OCR and IL-12p40, which may reflect reduced immune activation under conditions of higher oxidative metabolism. In contrast, a positive correlation between OCR and IL-7 was noted, suggesting a metabolic dependency on the secretion of this immune-modulating cytokine. These findings highlight the complex interplay between cellular metabolism and cytokine secretion in oesophageal adenocarcinoma and underscore the need for further studies to understand the biological implications of these correlations.

Elevated levels of angiogenic mediators such as VEGF-A and TSLP have been observed in tumours with higher oxidative phosphorylation levels [49,50]. Angiogenesis is crucial for tumour development and progression, as it facilitates nutrient delivery and waste removal from the tumour region [51]. Most anti-angiogenic agents approved for cancer treatment target VEGF signalling, given its key role in promoting angiogenesis [52]. In this study, we aimed to verify whether the antiangiogenic activity of P3 was maintained when conjugated with gold nanoparticles. Our findings demonstrated that AuNP-P3 significantly reduced the number of intersegmental vessels in zebrafish embryos by approximately 54%, comparable to the 58% reduction observed with P3 alone (Figure 5). In contrast, unconjugated gold nanoparticles did not exhibit antiangiogenic effects. These results indicate that the antiangiogenic effect of AuNP-P3 is mediated by P3 and is independent of the gold nanoparticles. Therefore, developing agents that target both angiogenesis, inflammation, and oxidative phosphorylation may represent a novel approach to overcoming radiation resistance in oesophageal adenocarcinoma.

In summary, our aim was to overcome the solubility and bioavailability limitations of P3 by generating a bioactive compound that could be used effectively in pre-clinical studies and, potentially, in clinical trials. Therefore, we conjugated P3 with gold nanoparticles (AuNP-P3).

The successful conjugation of P3 with gold nanoparticles addresses two major challenges as follows: enhancing the compound’s solubility and providing a carrier system for the targeted delivery of the drug. By ensuring that P3 remains active and effective when conjugated, we pave the way for its use in more advanced studies and applications. Further in vitro mechanistic studies will be essential to elucidate the uptake mechanisms and release kinetics of AuNP-P3. Investigating how AuNP-P3 enters OAC cells, whether through receptor-mediated endocytosis, passive diffusion, or other pathways, will provide critical insights into its intracellular delivery efficiency.

Moreover, the potential for direct tumour delivery, particularly in oesophageal adenocarcinoma patients undergoing neoadjuvant radio-chemotherapy, highlights a practical application for this formulation. Administering AuNP-P3 via endoscopy could achieve higher local concentrations at the tumour site, enhancing its efficacy while reducing systemic side effects. This novel approach represents a promising strategy to improve cancer treatment outcomes and warrants further exploration.

## 5. Conclusions

In conclusion, this study successfully developed AuNP-P3, a gold nanoparticle formulation of pyrazinib, to address the solubility limitations of P3 while retaining its radio-sensitizing properties. Across in vitro, ex vivo, and in vivo models, AuNP-P3 demonstrated radio-sensitization, the inhibition of oxidative phosphorylation and glycolysis, the modulation of inflammatory cytokines, and the suppression of angiogenesis. These findings highlight its potential to overcome radioresistance through dual anti-metabolic and anti-inflammatory mechanisms. Importantly, the nanoparticle formulation maintained P3’s bioactivity and offers targeted delivery advantages for clinical application in neoadjuvant radiochemotherapy for oesophageal adenocarcinoma. Future work will focus on the further validation and optimize the clinical translation of this promising therapeutic strategy, with the potential to improve clinical outcomes in patients facing this aggressive cancer.

## Data Availability

The data presented in this study are available upon request from the corresponding author due to an embargo period to allow for the commercialization of research findings.

## Supplementary Materials

The following supporting information can be downloaded at https://www.mdpi.com/article/10.3390/cancers16234007/s1, Supplementary Figure S1: Details of the gold nanoparticles orthogonal physical characterization by different techniques. Supplementary Figure S2: Details of the mitochondrial parameters measure in real-time in OE33P and OE33R cells in response to 10 µM AuNP-P3, AuNPs, and P3 treatment in the absence and presence of ionizing radiation. Supplementary Figure S3: Correlation plots showing correlations between the levels of released mediators and the metabolic rates of OE33P and OE33R cells in response to 10 µM AuNP-P3 treatment in the absence and presence of ionizing radiation. Supplementary Table S1: Summary of gold nanoparticle characterization.
