# Supplementary material for: Targeting Radiation Resistance in Oesophageal Adenocarcinoma with Pyrazinib-Functionalised Gold Nanoparticles
Source: Cancers (Basel). 2024 Nov 29;16(23):4007. doi: 10.3390/cancers16234007 (PMC11640662; doi:10.3390/cancers16234007)
Supplement: Supplementary file 1 [file cancers-16-04007-s001.zip › cancers-3291997-supplementary.pdf]

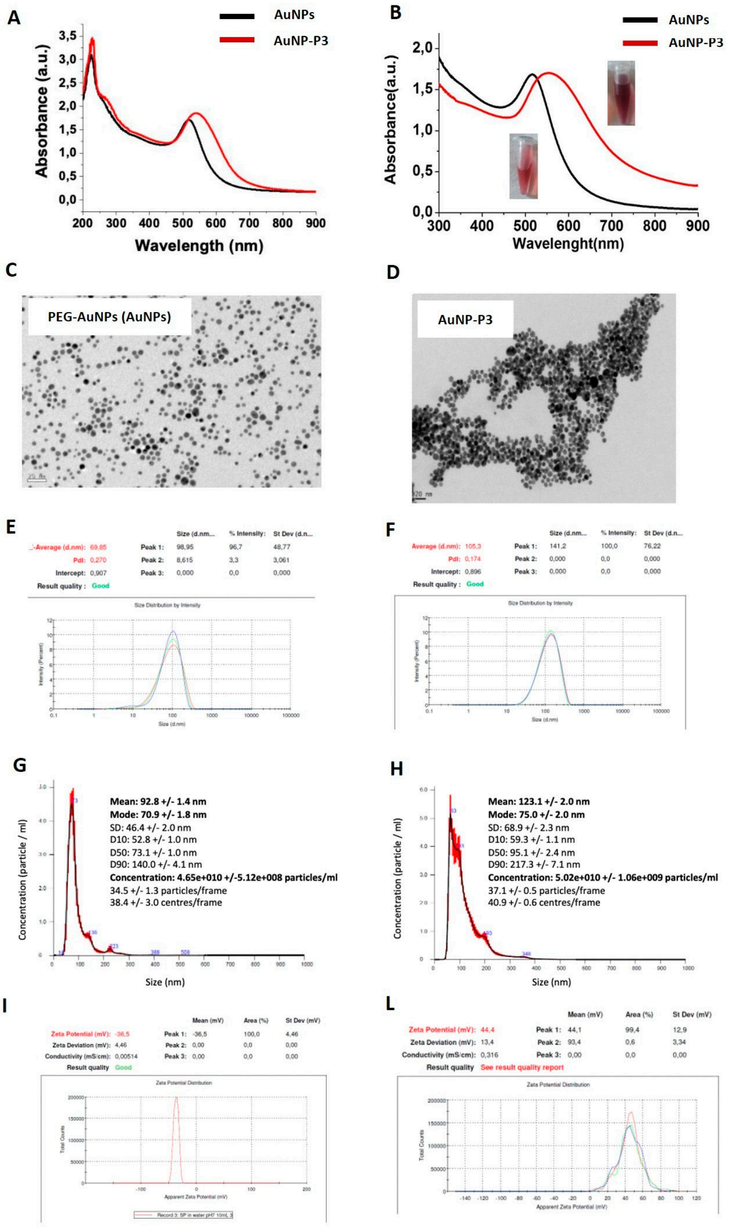

Supplementary Figure S1. Details of the gold nanoparticles orthogonal physical characterization by different techniques. (A,B) Typical UV-Vis Absorption Spectra acquired at room temperature at full range (A) and in the range 300–900 nm (B) for PEGnylated gold nanoparticle (AuNPs) and pyrazinib-functionalised gold nanoparticles (AuNP-P3). As the AuNP is the most stable particle since the PEGylation provide colloidal stability, the comparison with the AuNP-P3 spectra shows that the two formulations are stable and the only difference in the spectra is the P3-associated peak. (C,D) Transmission Electron Microscopy (TEM) images of AuNPs and AuNP-P3 at high magnification (scale bar 20nm). (E,F) Dynamic Light Scattering (DLS) plots as fingerprinting of AuNPs (E) and AuNP-P3 (F) hydrodynamic particle size distribution (as Z-average and main peaks), polydispersity index (PDI), and standard deviation. (G,H) Nanoparticle Tracking Analysis (NTA) size distribution plots as typical of AuNPs* (G) and AuNP-P3 (H). Showing the large size distribution of the AuNP-P3 compared to the AuNPs. (I,L) Zeta potential measurements for the two AuNPs and AuNP-P3 showing the different charges and charge distribution.

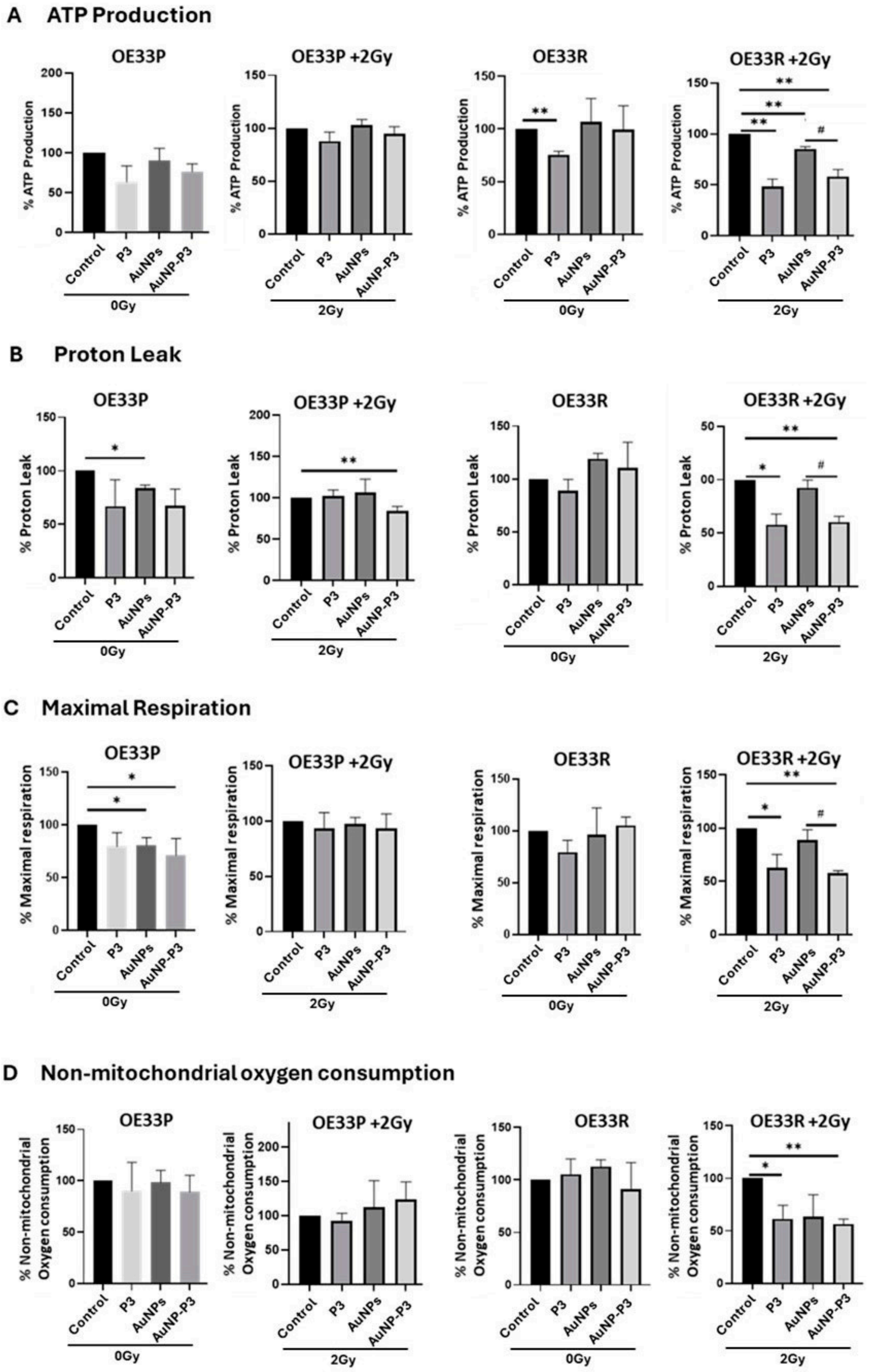

Supplementary Figure S2. Details of the mitochondrial parameters measure in real-time in OE33P and OE33R cells in response to 10 μM AuNP-P3, AuNPs, and P3 treatment in the absence and presence of ionizing radiation. OE33P (radiation sensitive cells) and OE33R (radiation resistant cells) were treated for 24 h with the compounds at 10 μM and subsequently irradiated at 2Gy IR. After 24 h, cells were used to measure metabolic rates in real-time using Seahorse Biosciences XFe24 analyser. (A) ATP production. (B) Proton leak. (C) Maximal respiration. (D) Non-mitochondrial oxygen consumption. Data are expressed as mean ± SEM. Statistical analysis was carried out using a paired *t*-test. * $p < 0.05$,** $p < 0.01$ (Compound vs. Control); # $p < 0.05$ (AuNP-P3 vs. AuNPs).

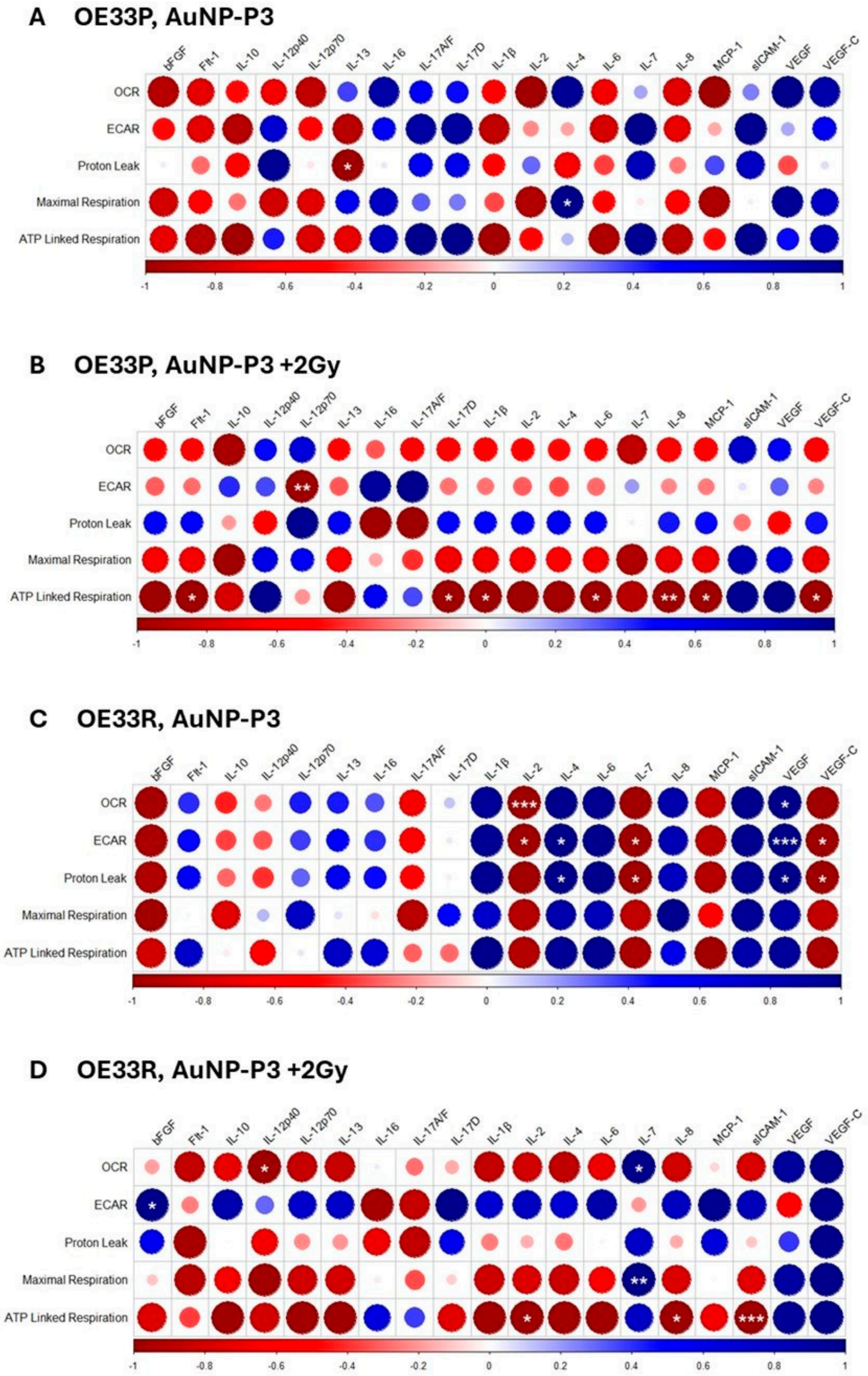

Supplementary Figure S3. Correlation plots showing correlations between the levels of released mediators and the metabolic rates of OE33P and OE33R cells in response to 10 μM AuNP-P3 treatment in the absence and presence of ionizing radiation. Correlation plot showing significant correlations between the levels of released mediators and the metabolic rates (Spearman correlation, blue indicates positive correlations and red indicates inverse negative correlations). The Holm–Bonferroni hoc correction was used to control for multiple comparison testing; * $p < 0.05$, ** $p < 0.01$, *** $p < 0.001$.

Supplementary Table S1. Summary of gold nanoparticle characterization. Data on PEG-AuNPs (or AuNPs) and AuNP-P3 as nominal size and standard deviation by Transmission Electron Microscopy (TEM). Hydrodynamic diameters measured by Dynamic Light Scattering (DLS) reported by Z-average and PDI and as the main peak and standard deviation as the outcome of n = 3 repeated measurements. Hydrodynamic diameters measured by Nanoparticle Tracking Analysis (NTA) reported as mean, mode, and standard deviation across the 6 repeated readouts. NTA particle concentration measured across the 6 repeated readouts. Finally, zeta potential measurements of both particles.

Particle type	TEM size ± st.dev [nm]	DLS size Z-avg [nm] (PDI)	DLS size main Peak ± st dev [nm]	NTA Mean [nm], Mode [nm], SD [nm]	NTA particle concentration [particle/ml]	Zeta Potential [mV]
**AuNPs**	4.6 ± 1.0	69.85 (0.270)	98.95 ± 48.77	92.8 ± 1.4, 70.9 ± 1.8, 46.4 ± 2.0	4.65e+010 +/- 5.12e+008	-36.5 ± 4.46
**AuNP-P3**	8.9 ± 1.6	105.3 (0.174)	141.2 ± 76.22	123.1 ± 2.0, 75.0 ± 2.0, 68.9 ± 2.3	5.02e+010 +/- 1.06e+009	44.4 ± 12.9
